# Nationwide mapping of terrestrial gamma radiation in South Korea using a car-borne survey system

**DOI:** 10.1038/s41598-025-34666-0

**Published:** 2026-01-08

**Authors:** Jaeho Jang, Jaewoo Park, Byung-Uck Chang, Yong-Jae Kim

**Affiliations:** 1 Korea Institute of Nuclear Safety, 62 Gwahak-Ro, 34142 Daejeon, Republic of Korea; 2 Department of Nuclear Engineering, Kyung Hee University, 1732 Deogyeong-daero, 17104 Yongin, Republic of Korea

**Keywords:** Terrestrial gamma radiation, Car-borne gamma-ray survey, Natural radionuclides, Radiation mapping, Environmental sciences, Physics

## Abstract

This study conducted a nationwide car-borne radiation survey to assess background radiation levels originating from naturally occurring radionuclides across South Korea. Two survey vehicles were developed, each equipped with NaI(Tl) detectors and high-pressure ionization chambers (HPICs), and deployed over a period of approximately four years. As a result, a total of 723,052 gamma-ray spectra and ambient dose rate measurements were collected. A post-processing tool incorporating an automatic peak-channel tracking algorithm and net count rate (CPS) calculation was developed to extract radionuclide-specific net count rates. These values, along with the dose rates, were converted into concentrations of equivalent uranium (eU), equivalent thorium (eTh), and potassium-40 (^40^K) as well as corrected ambient dose rates, using established conversion factors and shielding correction models from previous studies. The resulting geospatial database contains activity concentrations in soil and ambient gamma dose rates at each measurement points. The average concentrations were 51.2 Bq kg^-1^ for eU (range: 0.003–927 Bq kg^-1^), 58.9 Bq kg^-1^ for eTh (range: 0.02–556 Bq kg^-1^), and 877 Bq kg^-1^ for ^40^K (range: 21–3,135 Bq kg^-1^), with an average dose rate of 131 nSv h^-1^. The uncertainties represent standard deviations (k = 1). Four distribution maps were produced to visualize the spatial distribution of terrestrial radiation. The comprehensive and high-resolution data obtained through this study provide a quantitative foundation for assessing public radiation exposure and for informing national policy on the management of existing exposure situations.

## Introduction

Human beings are constantly exposed to radiation occurring in the natural environment, such as cosmic and terrestrial radiation. The average annual exposure dose from natural radiation for Koreans has been estimated at about 3.1 mSv, which is slightly higher than the global exposure dose of 2.4 mSv, both of which include spatially variable contributions from radon^1,2^. Meanwhile, the International Commission on Radiological Protection (ICRP) has recommended the implementation of optimization process using reference levels for existing exposure situations, including radon^3^. Radon (^222^Rn), an inert gas in the decay chain of ^238^U, is of particular concern because it is a major contributor of radiation exposure to the general population^4^. Radon levels vary significantly by region, and in Korea, elevated radon or thoron levels of detached houses were found to be associated with the concentration of parent nuclides in surface soil^3,5^. Due to its gaseous nature, radon is relatively more controllable through mitigation measures such as ventilation or installation of reduction systems, making it a priority for optimization. Radiation exposure in specific regions can be managed by designating them as radon-prone or radon-concerned areas as part of national mitigation policies.

The ICRP defined a radon-prone area as “one in which about 1% of dwellings had a radon concentration of more than ten times the national average value^6^.” The European Commission (EC) recommended using radon maps to establish measures against radon through the Radon Prevention And Remediation (RADPAR) Project^7^. However, if the indoor radon concentration decreases due to implementation of national radon policy, the area may no longer be classified as a radon-prone area. Nevertheless, the geological characteristics responsible for radon emissions remain unchanged, indicating that its radon potential risk is likely to persist. This study therefore focuses on establishing nationwide baseline data on terrestrial radioactivity to characterize such unchanging geological sources of radon. To this end, Korea Institute of Nuclear Safety (KINS) conducted a nationwide terrestrial radiation survey to evaluate the radiation background of natural radionuclides^8^.

The United States and Europe provide online maps containing various natural radiation information. United States Geological Survey (USGS) conducted the Airborne gamma-ray spectrometry (AGRS) survey as part of uranium resource evaluation, producing maps of the concentration of natural radionuclides (uranium, thorium and potassium) and gamma-ray absorbed dose in North America^9^. The Joint Research Centre (JRC) of the EC developed the European Digital Atlas of Natural Radiation including radionuclide concentrations in soil and terrestrial gamma dose on 10 km × 10 km grid to inform the public and scientific communities. These concentrations were analyzed, compared, and merged from two available geochemical databases, and the terrestrial gamma dose rate was obtained from monitoring results such as Geiger-Müller (GM) and proportional counters^10^.

Although South Korea has relatively small land area, its mountainous terrain makes AGRS challenging due to the need for altitude correction. Additionally, spectral smoothing caused by the high speed of aircraft is not suitable for obtaining precise data with improved resolution^11^. Furthermore, Korea lacks nationwide geochemical data on uranium (^238^U), thorium (^232^Th), and potassium (^40^K) concentrations. Therefore, a car-borne gamma-ray spectrometry survey, which employs a well-established methodology, was chosen for a radon potential mapping program^8,11^.

In parallel with this project, a study was conducted to measure radionuclides and radiation dose in soil precisely. A strong correlation was observed between the *in-situ* and laboratory high-purity germanium (HPGe) techniques with a linear correlation coefficient (R^2^) of 0.99 for ^226^Ra and ^232^Th and 0.975 for ^40^K, respectively^8^. NaI(Tl) detector in the car-borne survey system also showed a strong correlation with *in-situ* HPGe measurement. So, empirical formulas were derived to convert the count rate of each radionuclide of NaI(Tl) to activity concentrations (Bq kg^-1^ unit) in soil. Radiation dose rates were measured using a high-pressure ionization chamber (HPIC) within the same vehicle, and a correlation equation for vehicle’s shielding effect was developed by comparing in- and out-vehicle measurements on the same point in several places with different dose rates^8^.

In this study, a specially designed vehicle for the terrestrial radiation survey is introduced, along with the associated survey procedures and data post-processing methodology. The algorithm was designed to extract net count rate for each radionuclide from more than 700,000 NaI(Tl) Spectra obtained from the survey project. The count rates of ^238^U, ^232^Th and ^40^K were converted to activity concentrations using previously obtained empirical formulas. Also, the radiation dose rates measured with HPIC inside the vehicle were converted to the dose rate at 1 m above the ground considering the shielding effect of vehicle. Finally, maps of activity concentrations and radiation dose rate covering the entire territory of South Korea were produced using the above data.

The nationwide terrestrial radiation survey described in this study provided the fundamental dataset of activity concentrations (^238^U, ^232^Th, and ^40^K) that was subsequently used in our recent publication^12^. In that follow-up study, the data obtained here were further processed to estimate absorbed dose rates using Beck’s formula, visualize spatial dose distributions, and evaluate population-weighted effective doses and inter-nuclide correlations.

In contrast, the present paper focuses on the instrumental development, survey methodology, data acquisition, and post-processing algorithms that enabled the generation of this nationwide database. Together, these two studies represent consecutive stages of a single long-term project, where the current work provides the technical foundation and dataset for subsequent exposure assessment research.

## Experimental technique

### Development of environmental-radiation exploration vehicle (EREV)

**Fig. 1:**
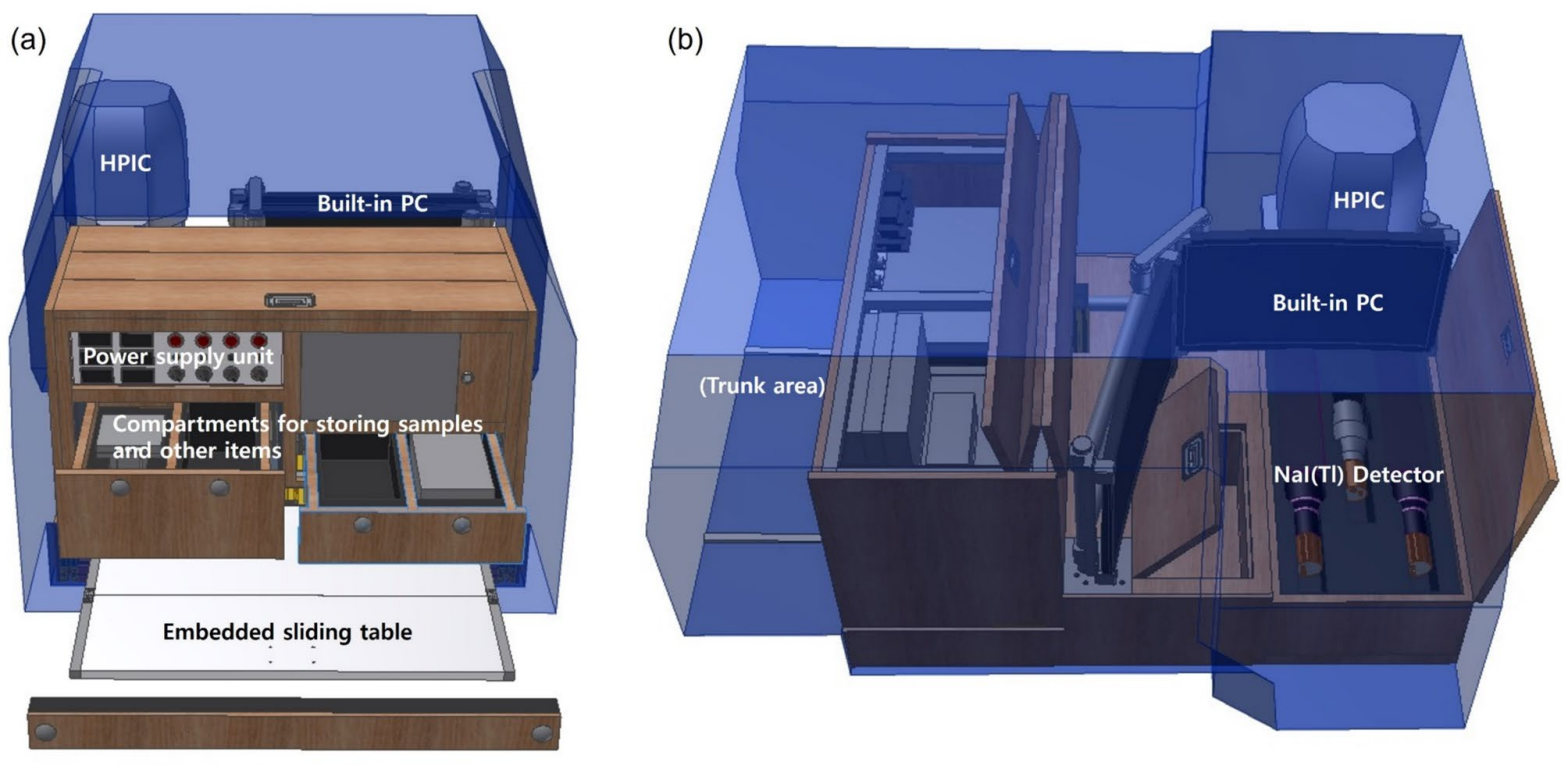
Schematic layout of the vehicle with rear-compartment modification and equipment arrangement: (**a**) rear view and (**b**) right-side view.

**Fig. 2:**
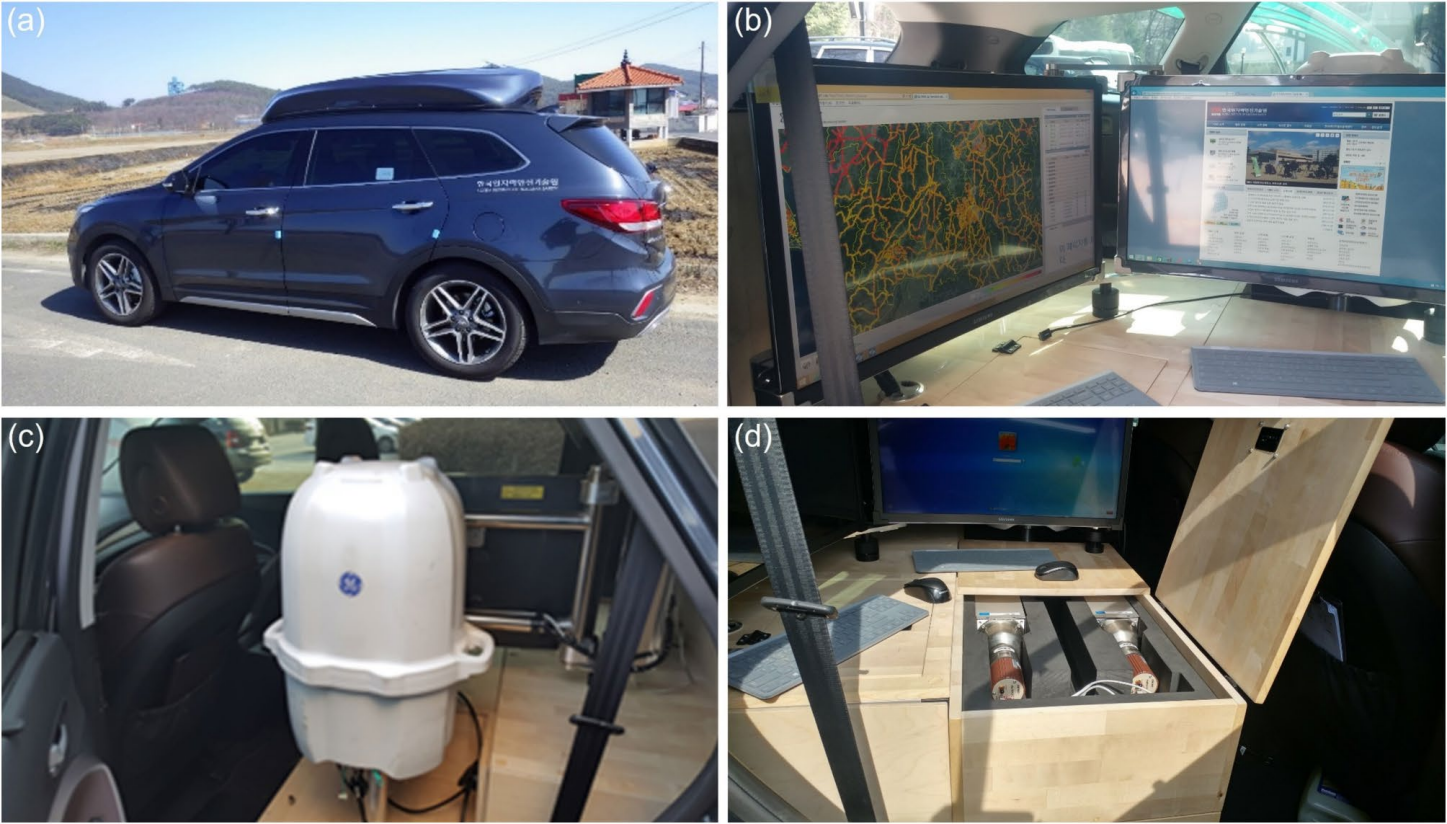
Developed Environmental-Radiation Exploration Vehicle (EREV): (**a**) exterior view, (**b**) operation PCs, (**c**) High-Pressure Ionization Chamber (HPIC), and ﻿(**d**) large-volume NaI(Tl) detectors.

Two Environmental-Radiation Exploration Vehicles (EREVs) were developed by converting standard sport utility vehicles (SUVs) into platforms permanently equipped with car-borne survey instruments and auxiliary systems. In order to acquire terrestrial gamma radiation spectra, a large volume NaI(Tl) detector (4″ × 4″ × 16″, SAINT-GOBAIN) used in natural radioelement mapping, and a multi-channel analyzer (MCA, 1024 channels, ORTEC Co.) were selected^8,11,13^. The resolution of NaI(Tl) detector is lower than HPGe, but it does not require cryogenic cooling, making it easy to use in the field for long periods of time and can be manufactured with high efficiency and large capacity. The ambient dose was measured with high-pressure ionization chamber (HPIC, 25.4 cm Φ, HPIC RS Detection, General Electric Co.) environmental radiation monitor, which is argon-filled at approximately 25 atm (~ 25 MPa). The EREVs were additionally equipped with Global Position System (GPS) terminal (GNSS, AscenKorea Co.), long-term evolution (LTE) wireless communication system, operation PCs with operating software (RadSearch Co.), and a system power supply module^8^. The schematic layout and photographs of the EREV and the on-board instruments in the rear compartment are presented in Figs. 1 and 2.

The gamma-ray spectrum, ambient dose rate and GPS coordinates during the survey are continuously measured, and each set of data is collected onto the PC via operating software. At this time, in order to secure high-density survey data for the entire territory of South Korea, the EREV’s speed was limited to 60 km/h or less^8^. Each measurement was performed at 10-s intervals, and the midpoint of 10-s driving segment was selected as the representative coordinate. Although this interval may reduce spatial resolution compared with stationary measurements, the car-borne system, operating at approximately 1 m above ground level, provides considerably higher spatial detail than airborne gamma surveys. The measurement time was determined based on the evaluation of energy windows for three nuclides, with relative uncertainties below 10% (maximum approximately 6%). The data were transmitted to the Korea Institute of Nuclear Safety (KINS) main server via the LTE network, and simultaneously fed back to the operating software to prevent duplication of survey routes^8^.

Surveys were not conducted during rain or snow, due to increase of dose rate by early precipitation and decrease of dose rate by the shielding effect of water or snow accumulated on the road. Road segments with unavailable GPS reception, such as those passing through structures like tunnels, were automatically excluded from the dataset. Data exclusion was not based on a predefined cut-off distance but determined by the availability of valid GPS signals. In addition, highways and automobile-only roads were excluded from the survey, as they are constructed with soil transported from other regions, making them unsuitable for reflecting the radiation characteristics of the area.

### Data processing

#### Technical challenges in data processing

Car-borne gamma-ray spectrometry with NaI(Tl) detector enables rapid and low-cost assessment of natural radioelement concentrations over large areas^8,11^. However, this requires processing the spectrum for each measurement point to extract the net counts for the uranium (^238^U), thorium (^232^Th), and potassium-40 (^40^K) nuclides. Three technical issues arise in this context.

**Fig. 3:**
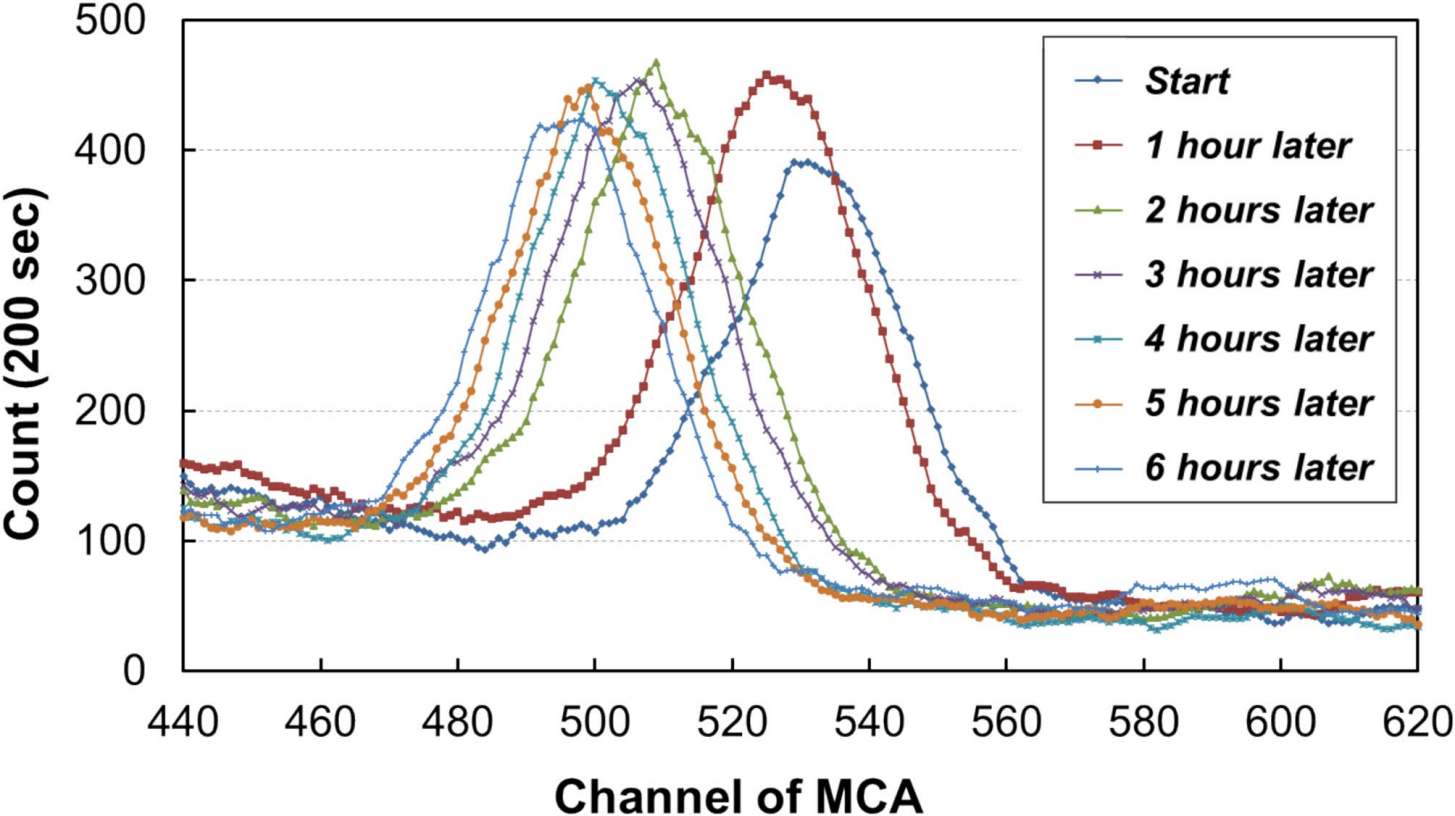
Example of temperature-induced channel drift over time in the gamma-ray spectrum of the NaI(Tl) detector (^40^K peak).

First, the temperature dependence of NaI(Tl) detector causes drift in the spectral channels^14,15^. As the survey is conducted over extended periods of time in uncontrolled environments, the acquired spectra exhibit peak drift correlated with temperature changes throughout the day (Fig. 3). Consequently, energy calibrations are necessary in the field to ensure data accuracy. However, performing frequent calibrations reduces the efficiency of the survey.

Second, each spectrum obtained in this survey has insufficient counts, making it challenging to accurately identify peak position of the target nuclide. This is due to the low count rate of the natural radiation environment and the short measurement time of 10 s per spectrum, in particular, at the region of very low natural radiation. In this measurement situation, background subtraction of the peak to calculate net counts, particularly for the ROI (region of interest) of ^214^Bi and ^208^Tl, which have significantly lower counts, can sometimes result in negative values.

Third, the number of spectra for this project is substantial. Assuming 8 h of measurement per day, 2,880 spectra are recorded per vehicle, resulting in approximately over 700,000 spectra collected throughout the entire survey. Consequently, processing each spectrum individually is impractical.

#### Development of a data post-processing tool

Considering the aforementioned challenges, we developed a data post-processing tool that continuously tracks the spectral peak-shifts of the NaI(Tl) and extracts the net count rate for each nuclide within the energy windows. This tool is applicable only to continuous time-series data collected by each survey vehicle. The data processing involves two main procedures: automatic peak-channel tracking and net CPS calculation for each nuclide.

**Fig. 4:**
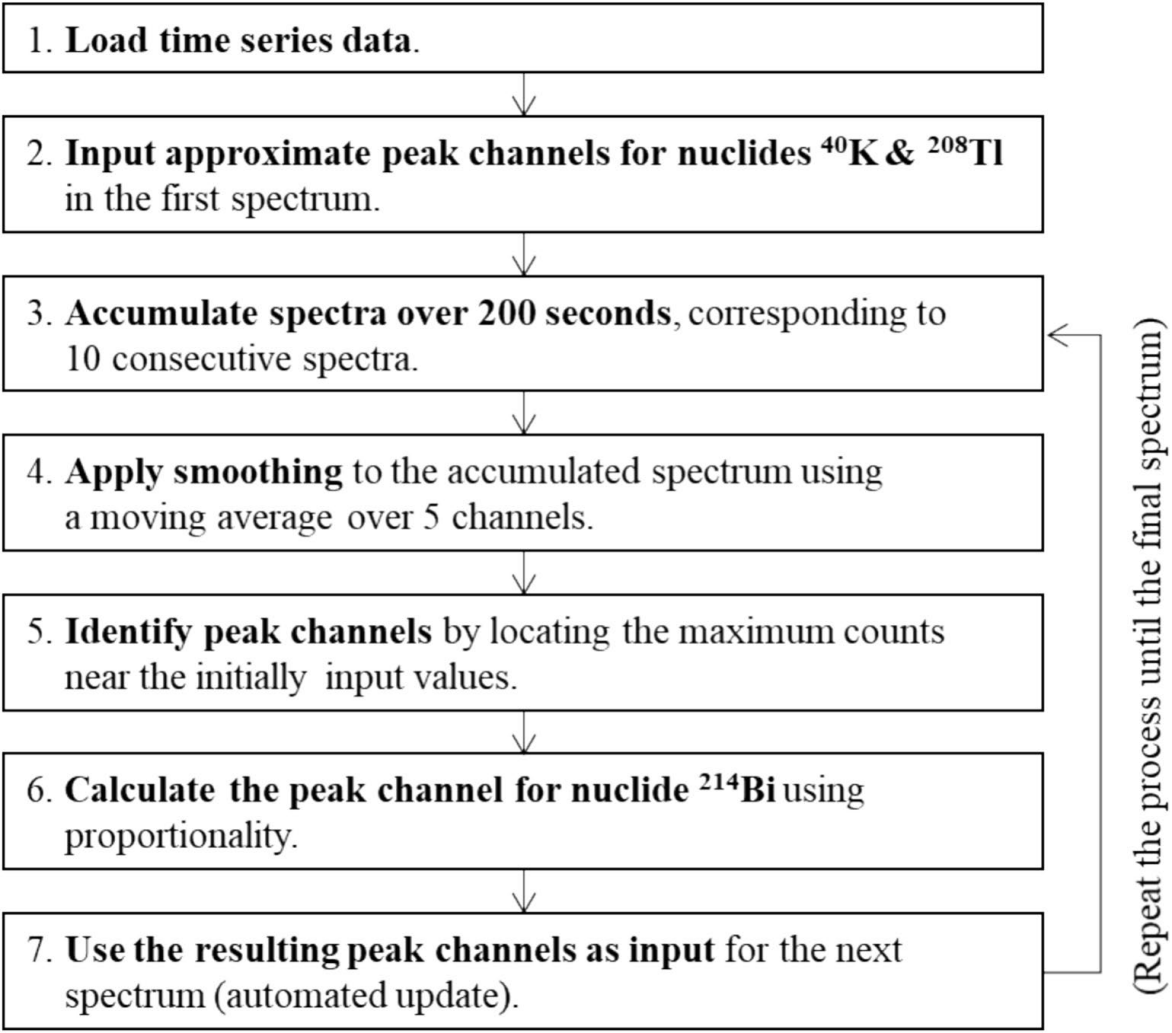
Automatic peak-channel tracking algorithm for the series data of NaI(Tl) gamma-ray spectra. (repeated until the final one, applying previously determined peak channels for the last 19 spectra where no subsequent data are available).

**Fig. 5:**
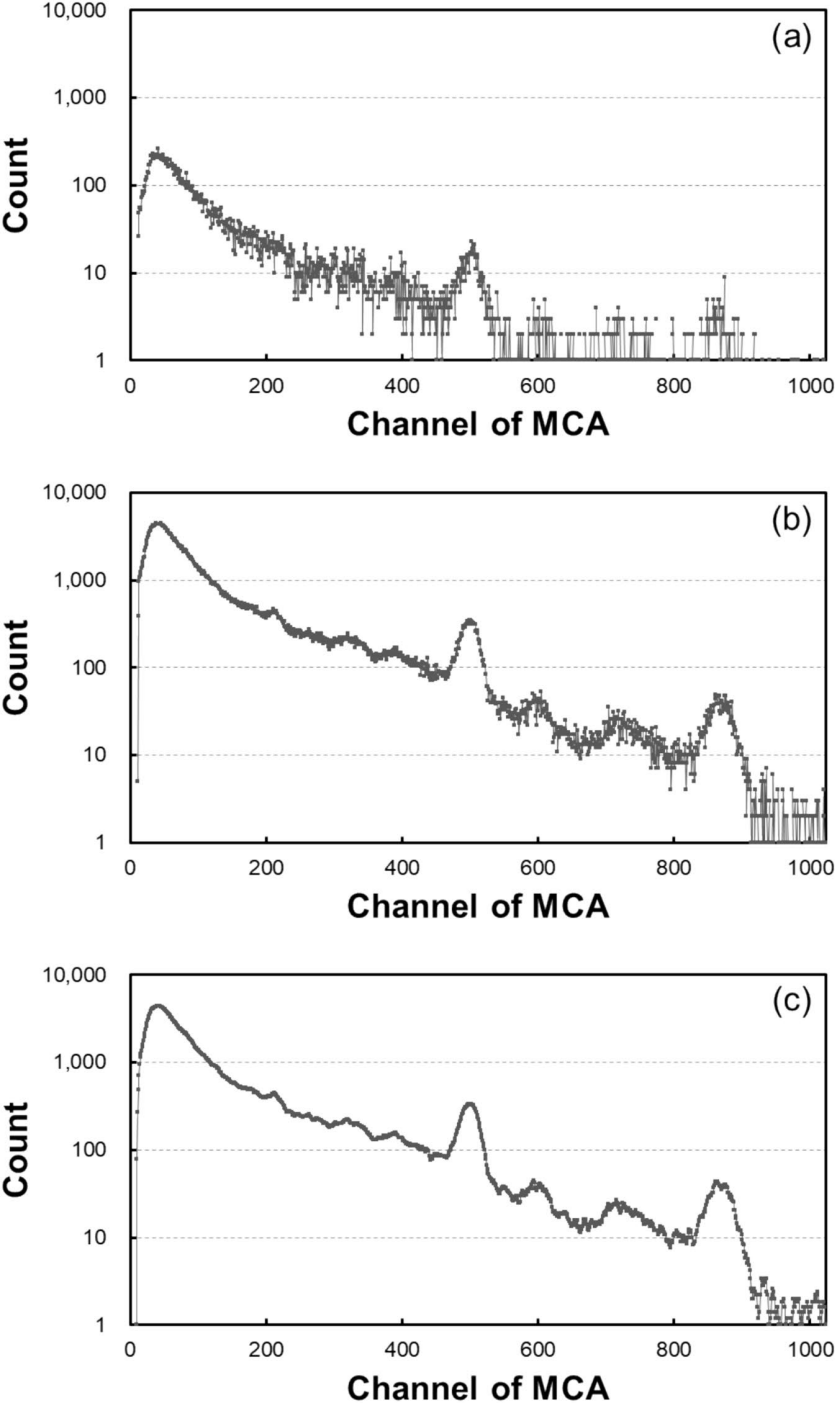
NaI(Tl) gamma-ray spectra: (**a**) measured with 10-s acquisition time, (**b**) accumulated over 200 s, and (**c**) smoothed using a moving average of five adjacent channels.

First, the automatic peak-channel tracking algorithm is shown in Fig. 4. This algorithm assumes little temperature change over short periods of time (less than 5 min), resulting in negligible spectral peak-shifts. However, each spectrum, measured only for 10 s, has low counts, making accurate peak-channel tracking challenging. To address this issue, a moving accumulation approach was adopted. Each spectrum, measured for 10 s, is denoted as *S*_*n*_. To determine the peak positions of *S*_*n*_, 20 consecutive spectra (*S*_*n*_ to *S*_*n*+*19*_) were summed. In addition, a smoothing method was employed by averaging the counts of each channel with those of the five adjacent channels, including itself (Fig. 5). The accumulated and smoothed spectrum was then used to identify the ^40^K and ^208^Tl (thorium series) peaks, while the less prominent ^214^Bi (uranium series) peak was calculated using a simple proportionality between the gamma-ray energy and the spectral channel for each nuclide. Once identified, the corresponding peak channels were applied to *S*_*n*_ for ROI-based count calculations. This process was repeated for each subsequent spectrum until the end of the dataset, with the last 19 spectra adopting the most recent determined peak positions.

By manually entering the approximate peak channels of the first spectrum of time-series dataset, the algorithm can accurately determine nuclide peaks, which are then used as automatic input values for peak-channel tracking in the subsequent spectrum. This process enables automatic determination of peak channels for all spectra. This method calibrates the energy of NaI(Tl) detector using the measurement results themselves, significantly reducing survey time by eliminating the need for repetitive and periodic field calibrations.

Second, the net count rate for each nuclide is calculated by subtracting the background count from the total count within the ROIs (Regions of Interest) of the three selected full energy peaks of the surveyed elements, and then dividing by the measurement time (livetime). The ROIs were determined using the proportionality between the range of the energy windows (1,370–1,570 keV for potassium; 1,660–1,860 keV for uranium; 2,410–2,810 keV for thorium) presented by^11^ and the channel of the spectrum, centered on peak channels identified by the peak-channel tracking algorithm above.

**Fig. 6:**
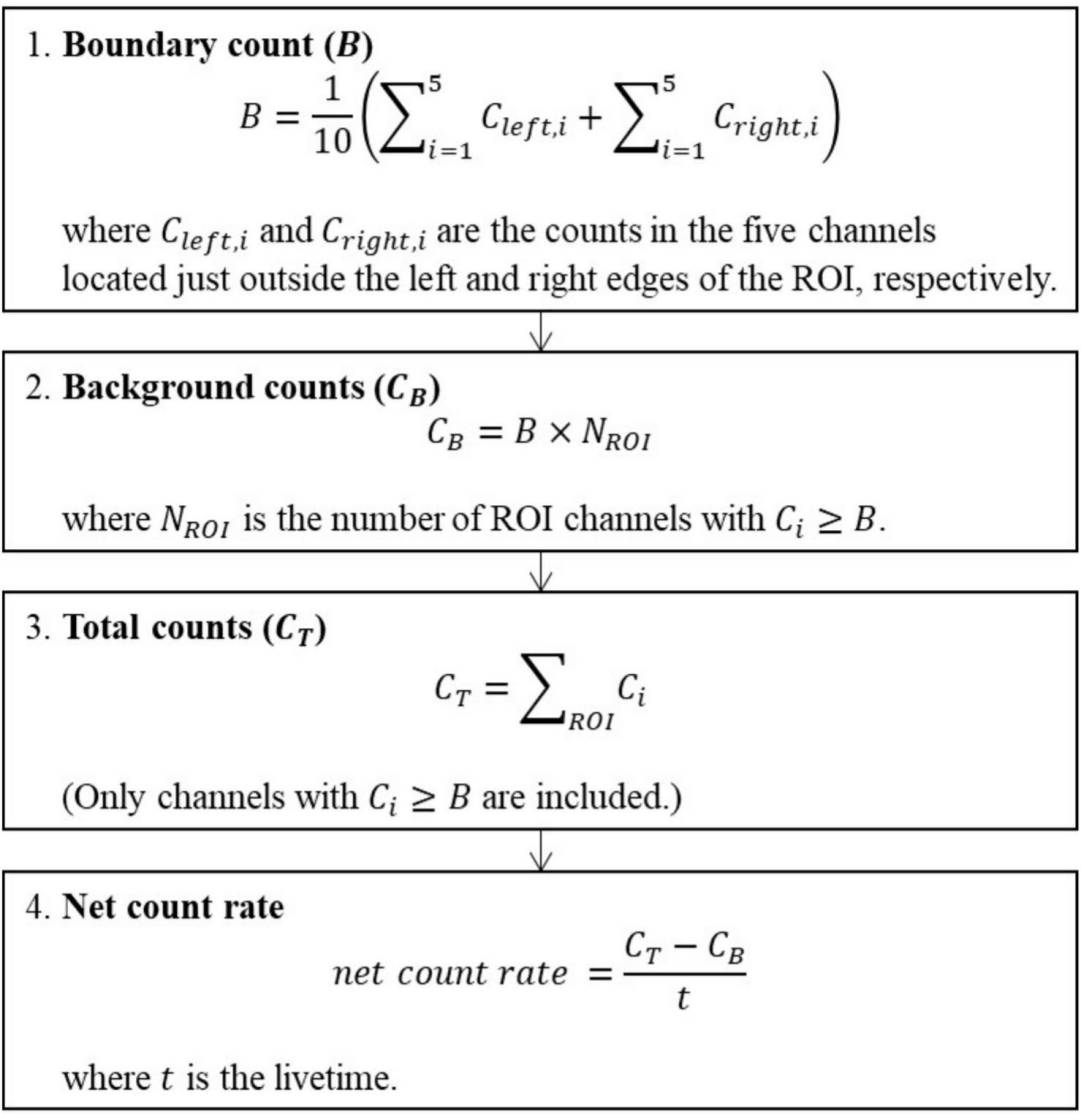
Procedure for calculating the net count rate.

The background count was estimated by multiplying the average count of channels located immediately outside the left and right edges of the ROI by the number of channels within the ROI. The boundary count was determined as the mean value of the 5 channels positioned just outside each side of the ROI. If the count in any channel within the ROI is lower than boundary count, that channel is excluded from the calculation. This criterion was applied pragmatically to prevent non-physical negative net count rates that may occur in low-statistics spectra, particularly in the uranium and thorium windows. Although this selective procedure may introduce a slight bias, its statistical impact on overall dataset is considered negligible, as it was applied only in geographically limited areas with extremely low count levels. The procedure for net CPS calculation is shown in Fig. 6.

Based on the above algorithm, a dedicated tool was developed to extract nuclide-specific net count rates from time-series spectral data collected through team-based surveys. By applying this tool, net count rate values were obtained for each GPS data point.

### Estimation of activity concentrations in soil and ambient dose rate

#### Determination of conversion factors of the car-borne measurement system for estimating nuclide-specific activity concentrations

In another study conducted in parallel as part of Korea’s radon potential mapping program, empirical formulas were derived to convert net count rates measured with NaI(Tl) detectors into activity concentrations for each natural radionuclide. In that study, flat, open areas with uniform activity concentration were selected through car-borne surveys, and gamma spectra were acquired using the car-borne NaI(Tl) detector in stationary mode at the central point. Around this center, five positions (one at the center, two at ± 5 m east–west, and two ± 10 m north–south) were measured using *in-situ* portable high-purity germanium (HPGe) detector. In addition, laboratory gamma-ray spectrometry was performed on soil samples collected from the same locations and compared with the *in-situ* HPGe results. Analysis of spectra acquired at the 11 locations confirmed a strong correlation between the *in-situ* HPGe and car-borne NaI(Tl) detectors. The resulting empirical formulas are as follows^8^.

1 $${Y}_{Ra}=0.087\bullet {X}_{Ra}+1.91 ({for}^{226}Ra)$$

2 $${Y}_{Th}=0.21\bullet {X}_{Th}+2.37({for}^{232}Th)$$

3 $${Y}_{K}=0.051\bullet {X}_{K}+3.50({for}^{40}K)$$

where *Y*_*Ra*_, *Y*_*Th*_, and *Y*_*K*_ are the net count rates of NaI(Tl) for uranium (via radium), thorium, and potassium, respectively, and *X*_*Ra*_, *X*_*Th*_, and *X*_*K*_ are the activity concentrations evaluated by *in-situ* HPGe. Using these formulas, the count rate for each radionuclide can be converted to activity concentrations (Bq kg^-1^ unit). Therefore, this method “is expected to be used to calibrate a car-borne measurement system equipped with a NaI(Tl) detector without previous characterization of the calibration site and background measurement of the detector^8^.”

#### Correction of car-shielding effect for ambient gamma dose rate

During car-borne surveys, the measurement equipment is mounted inside the vehicle, resulting in radiation shield due to the vehicle’s materials and other instrumentation. In the same study referenced previously^8^, the shielding effect was quantified by comparing high-pressure ionization chamber (HPIC) dose rates measured inside and outside the vehicle at locations with a wide range of radiation levels. A strong correlation (R^2^ = 0.999) was consistently observed between in-vehicle and out-vehicle measurements. The following shielding correction formula was derived:

$${D}_{in}=0.57\bullet {D}_{out}+18.80$$

where *D*_*in*_ is the dose rate measured inside the vehicle (nSv h^-1^), and *D*_*out*_ is the dose rate measured outside the vehicle (nSv h^-1^). The attenuation shielding factor was determined to be 0.57 (indicating 43% radiation attenuation), with a background radiation contribution from the vehicle’s materials of 18.8 ± 1.0 nSv h^-1^^8^.

## Results and discussion

### Terrestrial gamma radiation survey in South Korea

**Fig. 7:**
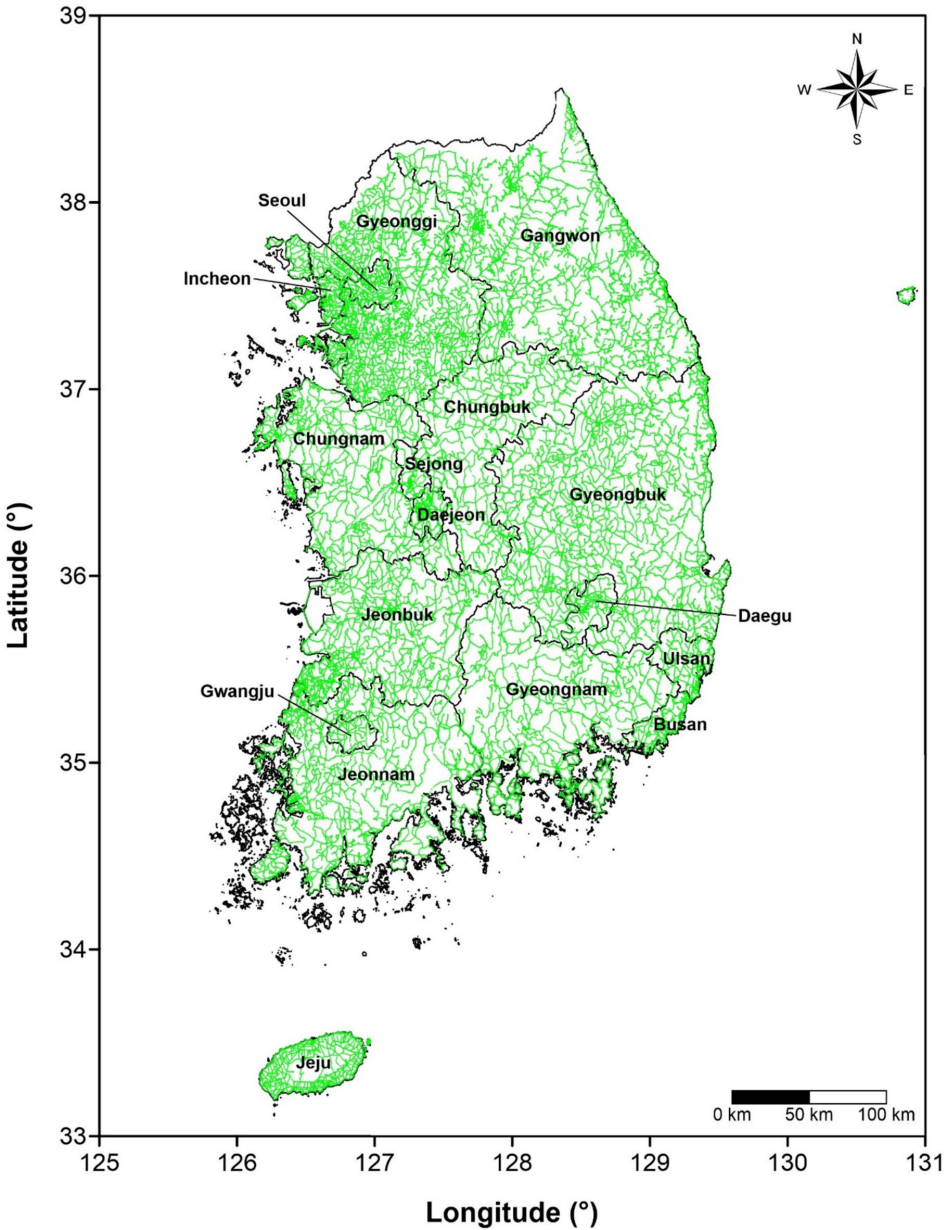
Nationwide survey route by the car-borne measurement system, reproduced from^12^, with permission from the copyright holder. The map was generated using Surfer® software (version 13.6.618, Golden Software, LLC; https://www.goldensoftware.com).

A terrestrial gamma-ray survey was conducted across the entirety of South Korea over a period of approximately four years (August 2013 – August 2017). Using the car-borne measurement system, NaI(Tl) gamma-ray spectra and ambient dose rate data were collected at 723,052 points. Assuming a constant vehicle speed of 60 km/h, the total survey path is estimated to span approximately 120,510 km. The regional and nationwide distribution of the survey (number of measured data and survey distance) are presented in Table 1 and Fig. 7, respectively.

**Table 1:** Number of measured data and survey distance.

City/Province	No. of spectra	Survey distance (km)*
Seoul	13,589	2,265
Busan	11,521	1,920
Daegu	7,586	1,264
Incheon	17,107	2,851
Gwangju	3,521	587
Daejeon	15,644	2,607
Sejong	3,405	568
Ulsan	10,609	1,768
Gyeonggi	115,479	19,247
Gangwon	120,132	20,022
Chungbuk	37,160	6,193
Chungnam	46,569	7,762
Jeonbuk	52,293	8,716
Jeonnam	69,873	11,646
Gyeongbuk	125,717	20,953
Gyeongnam	49,982	8,330
Jeju	22,865	3,811
Total	723,052	120,510

* Assumed constant vehicle speed of 60 km/h.

### Establishment of a database for soil concentration and ambient gamma dose rate

All NaI(Tl) spectral data acquired through the car-borne survey were processed using the dedicated post-processing tool to extract nuclide-specific net count rates. Each net count rate was then converted to the activity concentration in soil using the three empirical formulas. Additionally, the dose rates measured inside the vehicle with HPIC were corrected to external ambient gamma dose rates using the shielding correction formula. As a result, a database comprising over 700,000 datasets was established, including longitude, latitude, activity concentrations (for ^238^U, ^232^Th, ^40^K), and ambient dose rate. ^238^U and ^232^Th were expressed as equivalent uranium (eU) and equivalent thorium (eTh), since they were not analyzed directly but derived from the gamma rays emitted by their daughter nuclides (^214^Bi and ^208^Tl) in soil and rocks. To enhance the reliability of this conversion, an additional correction for radon loss (~ 15%) from surface soils was applied to ^238^U. This value was derived from a comparison between *in-situ* portable HPGe and laboratory gamma-ray spectrometry results, where the soil samples were carefully sealed in plastic containers and stored for one month to prevent radon loss. The observed difference, corresponding to expected radon escape under ambient conditions, confirmed a strong correlation (R^2^ = 0.99) between the two measurement techniques^8^.

For the entire territory of South Korea, the average activity concentrations were found to be 51.2 ± 34.9 Bq kg^-1^ for eU, 58.9 ± 33.0 Bq kg^-1^ for eTh, and 877 ± 261 Bq kg^-1^ for ^40^K, where the uncertainties represent standard deviations (k = 1). The concentration ranges were 0.003—927 Bq kg^-1^ for eU, 0.02—556 Bq kg^-1^ for eTh, and 21—3,135 Bq kg^-1^ for ^40^K. The ambient gamma dose rate ranged from 34.5 to 508 nSv h^-1^, with an average value of 131 ± 36 nSv h^-1^ (k = 1). These values represent simple arithmetic averages based on the overall survey data of South Korea. Therefore, when weighted by factors such as regional area or population, the results may differ slightly. The ambient gamma dose rate, directly measured using HPICs, partly includes contributions from cosmic rays and other external sources in addition to terrestrial radiation. According to calculations using the CARI-6 program, the average cosmic radiation dose rate at ground level in South Korea has been reported to be approximately 33.4 nSv h^-1^ [1]. In addition. Hassan [8] derived a value of 37.6 nSv h^-1^ by comparing measured dose rates with those calculated from activity concentrations using the Beck’s formula based on UNSCEAR-reported conversion factors^16,17^.

### Compilation of terrestrial radiation maps for South Korea

**Fig. 8:**
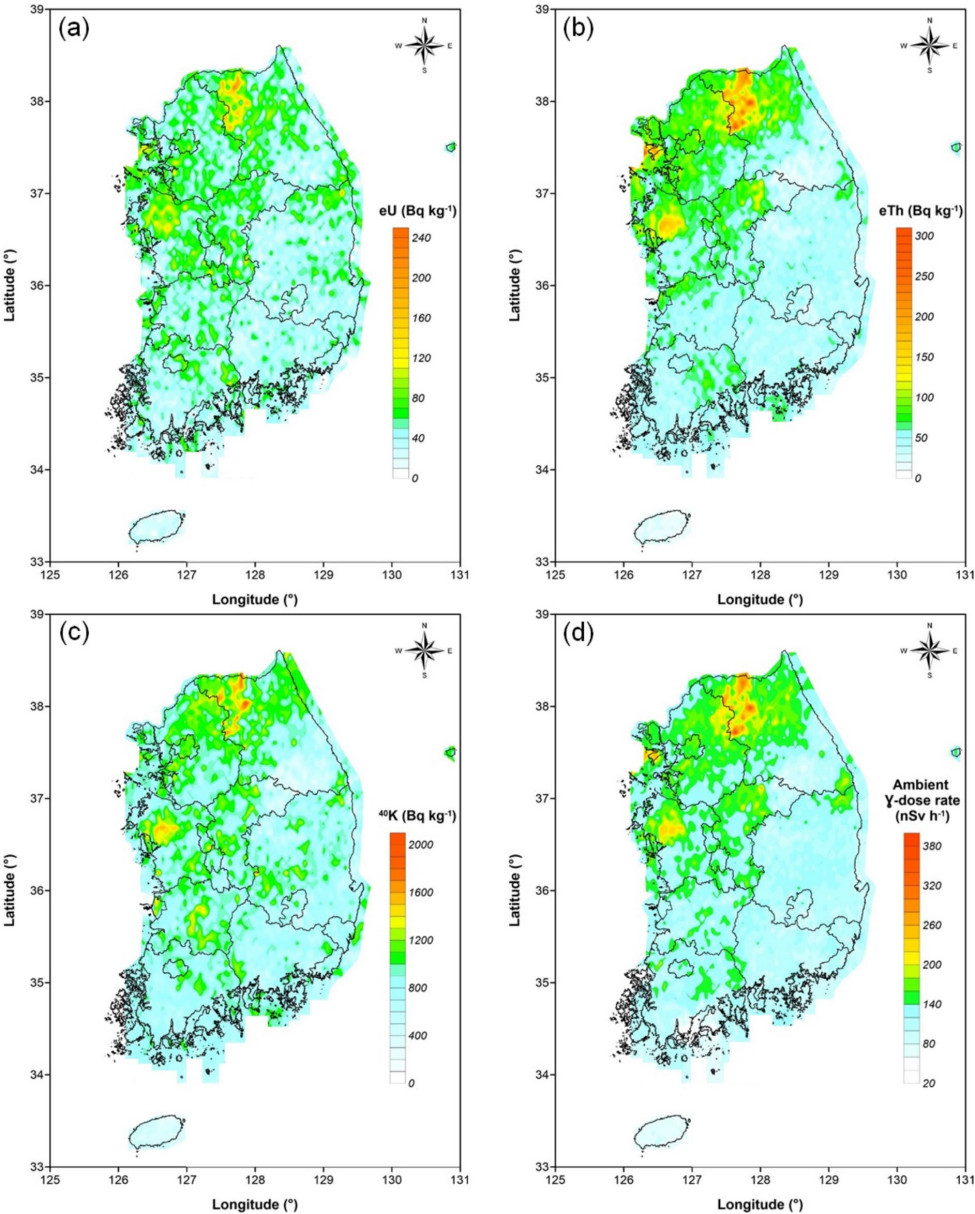
Maps of the spatial distribution of (**a**) equivalent uranium (eU), (**b**) equivalent thorium (eTh), and (**c**) potassium (^40^K) concentrations, as well as (**d**) ambient gamma dose rate, generated using Surfer® software (version 13.6.618, Golden Software, LLC; https://www.goldensoftware.com).

Using the established database, four distribution maps of natural radionuclides and gamma radiation (eU, eTh, ^40^K, and ambient dose rate) across the entire territory of South Korea were produced. The measurement values at each GPS coordinate were processed using Surfer^Ⓡ^ software, which mapped the data onto a regular grid through kriging interpolation, accounting for statistical correlations with surrounding points. The resulting distribution maps are presented in Fig. 8. The spatial distribution map of ambient gamma dose rates revealed relatively elevated levels in areas such as Chuncheon and Hwacheon (Gangwon), Yeongjong-do (Incheon), and Hongseng (Chungnam), while lower levels were observed in regions such as Jeju Island, Jecheon and Danyang (Chungbuk), and Yeongwol (Gangwon). Going forward, the equivalent uranium (eU) concentration maps, for instance, may potentially serve as proxy source terms for radon emitted from the ground surface.

## Conclusion

This study conducted a nationwide car-borne radiation survey across South Korea to assess background radiation levels from naturally occurring radionuclides. Two survey vehicles (EREVs) were developed, each equipped with NaI(Tl) detectors and HPICs for simultaneous gamma-ray spectrometry and dose rate measurements. Over a four-year period (August 2013 – August 2017), high-resolution measurements were obtained at 723,052 points nationwide.

A post-processing tool was developed to extract radionuclide-specific net count rates from each spectrum using automatic peak-channel tracking and net CPS calculation algorithm. These values were converted to eU, eTh, ^40^K concentrations, and corrected dose rates using established conversion factors and shielding corrections^8^.

As a result, a comprehensive geospatial database was established, containing activity concentrations in soil and ambient gamma dose rates across South Korea. Average activity concentrations were 51.2 Bq kg^-1^ for eU, 58.9 Bq kg^-1^ for eTh, 877 Bq kg^-1^ for ^40^K, with an average dose rate of 131 nSv h^-1^. Furthermore, four geospatial distribution maps of terrestrial radiation (eU, eTh, ^40^K, and ambient dose rate) were generated from the database, providing visual representation of the national radiation profile.

The dataset presented in this study, obtained and evaluated using consistent methodology, comprehensively covers nearly the entire territory of South Korea and, in terms of both spatial coverage and sample size, is sufficient to characterize the distribution of terrestrial radiation in inhibited areas of the country. However, as regions inaccessible by vehicles, such as mountainous areas, were excluded, the representativeness of the data for the entire territory may still be improved. By conducting further analyses subdivided by region, this database can serve as a quantitative reference for estimating radiation exposure both nationwide and at the administrative district level in the future. Additionally, by comparing the results with various geological and environmental features, such as bedrock types, soil composition and fault-line distribution, the dataset can serve as a proxy source term for estimating indoor radon levels. Combined with other relevant factors, including housing characteristics and climatic conditions, it can provide a more comprehensive basis for assessing radon-related exposure risks and supporting the designation of radon-prone areas.

## Data Availability

The dataset generated during the current study are not publicly available due to national-level sensitivity regarding regional variations in radiation levels, but are available from the corresponding author on reasonable request.
